# New aspects in cardiorenal syndrome and HFpEF

**DOI:** 10.1093/ckj/sfac133

**Published:** 2022-05-06

**Authors:** Ana Belén Méndez, Maria Antonieta Azancot, Aleix Olivella, María José Soler

**Affiliations:** Cardiology Department, Vall d'Hebron University Hospital, Vall d'Hebron Research Institute (VHIR), Barcelona, Spain; Nephrology Department, Vall d'Hebron University Hospital, Universitat Autònoma de Barcelona, Nephrology and Kidney Transplant Research Group, Vall d'Hebron Research Institute (VHIR), Barcelona, Spain; Cardiology Department, Vall d'Hebron University Hospital, Vall d'Hebron Research Institute (VHIR), Barcelona, Spain; Nephrology Department, Vall d'Hebron University Hospital, Universitat Autònoma de Barcelona, Nephrology and Kidney Transplant Research Group, Vall d'Hebron Research Institute (VHIR), Barcelona, Spain

**Keywords:** AKI, cardiorenal syndrome, chronic renal failure, diuretics, heart failure

## Abstract

Cardiorenal syndrome (CRS) is a complex disease in which the heart and kidneys are simultaneously affected, and subsequently, the malfunction of one organ promotes the deterioration of the other. Heart failure (HF) with preserved ejection fraction (HFpEF) is the most common form of HF. The pathophysiology of CRS is not well known and several mechanisms have been proposed. An elevation of central venous pressure seems to be one of the key points to consider, among others such as an increase in intraabdominal pressure. Several diagnostic tools have been identified to establish the diagnosis of CRS in patients with HFpEF. Currently, the availability of biomarkers of renal and cardiac injury, the use of pulmonary ultrasound, the monitoring of the size of the inferior vena cava and the study of the renal venous pattern offer a new dimension in accurately diagnosing and quantifying organ damage in CRS. Beyond the symptomatic treatment of congestion, until recently specific therapeutic tools for patients with CRS and HFpEF were not available. Interestingly, the development of new drugs such as the angiotensin/neprilysin inhibitors and sodium-glucose cotransporter-2 (SGLT-2) inhibitors offer new therapeutic strategies with potential benefits in reduction of cardiorenal adverse outcomes in this population. Randomized clinical trials that focus on patients with HFpEF are currently ongoing to delineate optimal new treatments that may be able to modify their prognosis. In addition, multidisciplinary teamwork (nephrologist, cardiologist and nurse) is expected to decrease the number of visits and the rate of hospitalizations, with a subsequent patient benefit.

## INTRODUCTION

Cardiac and renal diseases are both common and often coexist in the same patient, interacting and sharing similarities in their pathophysiologic mechanisms. Cardiorenal syndromes (CRS) are broadly defined as disorders of the heart and kidneys whereby acute or chronic dysfunction in one organ may induce acute or chronic dysfunction of the other [1, 2]. Chronic kidney disease (CKD) has a high global prevalence estimated between 9 and 15% [3, 4] and currently, the sum of individuals with CKD, acute kidney injury (AKI) and those on renal replacement therapy (RRT) exceeds 850 million people worldwide [5, 6]. Among patients with CKD defined as glomerular filtration rate (GFR) <60 mL/min/1.73 m^2^, cardiovascular disease is the leading cause of death [7] and renal dysfunction is one of the most important risk factors predicting mortality and poor outcomes in heart failure (HF) patients, with more than double risk of death if CKD is present [8, 9]. HF is a major clinical and public health problem, the current worldwide prevalence is estimated at 64.34 million cases (8.52 per 1000 inhabitants, 29% of which are mild, 19% moderate and 51% severe HF) [10]. Estimating the prevalence of HF in the CKD population has been challenging [11].

**FIGURE 1:**
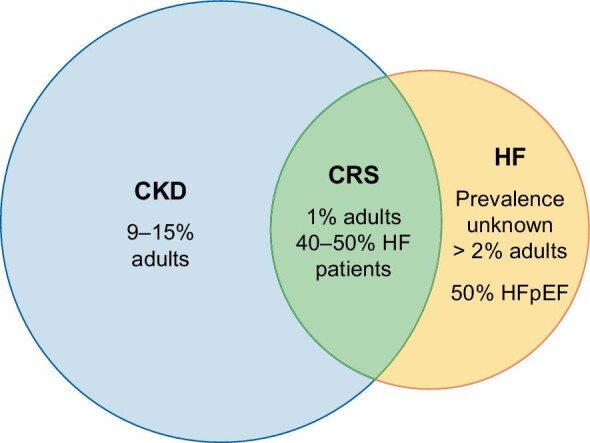
Estimated prevalence of CKD. CKD, chronic kidney disease; HF, heart failure; CRS, cardiorenal syndrome; HFpEF, heart failure with preserved ejection fraction.

**FIGURE 2:**
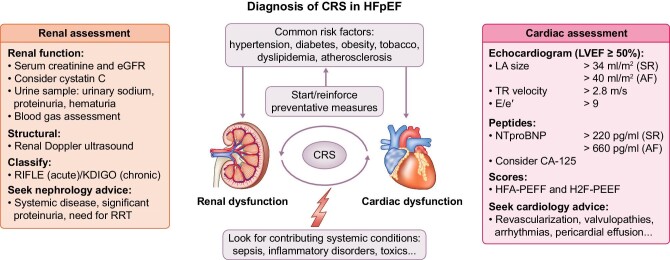
Suggested algorithm for CRS diagnosis. HfpEF, heart failure with preserved ejection fraction; CRS, cardiorenal syndrome; eGFR, estimated glomerular filtration rate; LA, left atria; TR, tricuspid regurgitation; SR, sinus rhythm; AF, atrial fibrillation; NTproBNP, N-terminal prohormone of brain natriuretic peptide; CA-125, cancer antigen 125.

Almost 30% of CKD Medicare patients have HF, compared with just 6% of Medicare patients without CKD [12, 13], and among dialysis patients the incidence of HF is 7% per year [14]. In other series, about 40%–50% of patients with HF have coexisting chronic renal dysfunction [15]. Figure 1 shows the estimated prevalence of CKD. Given the high burden of both HF and CKD, their complex interaction and challenging management, along with the prognostic implications regarding comorbidity and mortality, a comprehensive approach to CRS is mandatory, especially in the HFpEF population where HF diagnosis has been more evasive for years. Here, we propose a comprehensive review of the pathophysiology, diagnostic workup and management of CRS in HFpEF (Fig. 2).

## DEFINITION OF CRS

CRS is a complex disease in which the heart and kidneys are affected simultaneously. There is an imbalance between the two, in which the malfunction of one organ promotes, consequently, the deterioration of the other. Depending on this, as well as the onset, acute or chronic, there is a classification that differentiates five types of CRS. The origin of the CRS can be the heart, the kidneys or even both, and its onset can be acute or chronic, based on which a classification has been established [16]. Thus, type 1 refers to the AKI caused by acute cardiac disease; type 2 to CKD caused by chronic heart disease; type 3 to the heart dysfunction caused by the acute worsening of kidney function; type 4 to cardiac disease determined by CKD; and finally, type 5 is characterized by a simultaneous injury of the heart and kidneys caused by systemic diseases [1].

## HEART FAILURE WITH PRESERVED LVEF

The diagnosis of HFpEF is challenging. These patients generally do not have a dilated left ventricle (LV), but they often show a thickened LV or increased myocardial mass and/or increased left atrial size as a sign of augmented filling pressures. Most have additional ‘evidence’ of impaired LV filling or suction capacity, also referred to as diastolic dysfunction, which is generally accepted as the likely cause of HF in these patients [17].

Two score-based algorithms (H2FPEF and HFA-PEFF) have been developed for HFpEF diagnosis [18, 19]. The first relies on clinical characteristics and echocardiogram findings and the latest uses biomarkers, functional and morphological parameters from an echocardiogram to estimate HF probability. Both have their limitations and perform differently depending on the scenario. For example, in H2FPEF score, obesity and older age, both of them highly prevalent in CKD, lead to a high estimated probability of HF and in HFA-PEFF, natriuretic peptides (NP) might be elevated in CKD regardless of HF status. Recently, the latest HF Guidelines [17] define the HFpEF as those patients with normal LVEF, typically considered as ≥50%, symptoms and signs of HF and objective evidence of cardiac structural and/or functional abnormalities consistent with the presence of LV diastolic dysfunction/raised LV filling pressures, including raised NP. As a result of increasing awareness and clarification of diagnostic criteria [20], there has been an increase in the rate of diagnosed patients and nowadays HFpEF is the most common form of heart failure, comprising more than 50% of all patients [21], and the prevalence is still increasing over recent years [22]. HFpEF is associated with comorbidities including older age, obesity, CKD, atrial fibrillation and hypertension. These conditions, especially in patients with CKD, may exacerbate sodium and fluid retention leading to congestion [23].

In HFpEF, there is a disproportionate increase in intracavitary diastolic pressure for any rise in volume. This leads to a retrograde increase in left atrial and pulmonary venous pressure, which causes symptoms and signs of pulmonary venous congestion. Postcapillary pulmonary hypertension resulting from increased pulmonary venous pressure may precipitate right heart failure. Left ventricular stroke volume and cardiac output may also decline because of decreased end-diastolic volume (preload dependent) [24].

## PATHOPHYSIOLOGY OF CRS IN THE PATIENT WITH HFpEF

### Intraabdominal and central venous pressure elevation

HF, and therefore also patients with HFpEF, is characterized by an elevation in central venous pressure (CVP), which attenuates the gradient through the glomerular capillary network, leading to decreased renal perfusion and lower glomerular filtration. An increased CVP also produces a cascade of elevated intraabdominal pressure, pulmonary hypertension and endothelial dysfunction. Rising renal venous pressure limits urine formation and renal blood flow more than a reduction in arterial pressure [25].

The concept that venous congestion, not arterial blood flow, is an important mediator of cardiorenal failure is supported by the findings of the Evaluation Study of Congestive Heart Failure and Pulmonary Artery Catheterization Effectiveness (ESCAPE) trial, in which only baseline right atrial pressure, not arterial blood flow, correlated with baseline serum creatinine [26]. In accordance with this, Mullens *et al.* [27] also demonstrated that in patients with advanced HF admitted for HF decompensation, worsening renal function is commonly observed despite haemodynamic improvements with intensive medical therapy.

### Renin-angiotensin-aldosterone axis and renal dysfunction

Classic studies in patients with decompensated HF showed relative preservation of the GFR despite having reductions in renal blood flow [28]. This could be, in part, explained by the reduction of renal blood flow, an elevation of renin levels, which leads to vasoconstriction of the efferent arteriole, conditioning an increase in the filtration fraction derived from high intraglomerular pressure. Due to the fact that in decompensated HF these elevated renal venous pressures are perpetuated, there is an activation of the renin-angiotensin-aldosterone system (RAAS) and neurohormonal system, which causes vasoconstriction of the afferent arteriole to ensure renal blood flow that ultimately produces a reduction in the GFR. In addition, there is an increased reabsorption of sodium and water in the proximal tubules to maintain effective plasma volumes, eventually resulting in oliguria and worsening of congestion [29]. This is the same mechanism that results in the worsening of kidney function when RAAS inhibitor drugs are started in these patients.

Chronic RAAS activation in HF may contribute to cell hypertrophy, inflammation, apoptosis, fibrosis and oxidative stress in both the heart and kidneys. In addition, it impairs mitochondrial function and increases mitochondrial-derived oxidative stress, which in turn can lead to kidney damage and sodium and water retention, contributing to worsening congestion [30].

### Other mechanisms

Comorbidities in HFpEF such as obesity, hypertension and diabetes mellitus, among others, produce a systemic pro-inflammatory state, which causes coronary microvascular endothelial inflammation, and it is known that endothelial dysfunction is the primary pathophysiologic abnormality in HFpEF. Furthermore, coronary microvascular dysfunction has been proposed to be a novel mechanism underlying the pathogenesis of HFpEF [31]. Pulmonary hypertension is widely prevalent in patients with HFpEF [32], and those with right heart failure have a poor prognosis. Additionally, the neurohormonal and sympathetic activation in these patients promotes CKD [33].

Chronotropic incompetence has also emerged as a crucial pathophysiological mechanism in HFpEF and might be related to autonomic dysfunction with decreased baroreflex sensitivity and increased sympathetic stimulation [34]. A recent Spanish trial suggests that deprescribing beta-blockers in patients with HFpEF and chronotropic incompetence seems a plausible intervention to improve functional capacity [35].

## DIAGNOSIS OF CRS FROM BIOMARKERS TO CLINICAL IMAGE TOOLS

Diagnosis of HF requires the presence of signs and symptoms, along with evidence of a structural or functional cardiac abnormality [17] and in CRS, this requirement extends to the heart and kidneys. As signs and symptoms of volume overload and increased filling pressures are commonly found in both of them, to date several diagnostic tools can help to establish the diagnosis, including biomarkers, non-invasive imaging modalities or invasive haemodynamic monitoring, which are summarized in the following sections (Fig. 2).

### Biomarkers in CRS

The use of biomarkers has expanded during the last decades in HF and CKD, being used as diagnostic, prognostic and therapy response tools. This field has been rapidly expanding and many biomarkers have been proposed such as B-type natriuretic peptide (BNP), Troponin I and T(cTnI, cTnT) and Carbohydrate antigen 125 (CA125) [1, 36].

BNP and its inactive cleavage protein N-terminal pro-B-type natriuretic peptide (NTproBNT) are well-established markers of myocardial stretch widely used in HF management, with diuretic, natriuretic, vasodilation and other cardioprotective properties. However, in the presence of CKD, NP remain elevated, not simply because of a diminished clearance but as a counter-regulatory response from the heart to the kidneys [37]. In patients with CKD, adjusted BNP and NTproBNT cut-points are not available for HF diagnosis and for this reason its use in the setting of advanced CKD is controversial, especially in patients on dialysis. In addition, NP are less sensible in HFpEF compared with HFrEF and more strict cut-offs have been proposed, which limits its usefulness even more in the scenario of altered kidney function [19]. Nevertheless, high NP should not be ignored in the setting of renal dysfunction, as clearly elevated levels point toward cardiac disease. Future work is needed to establish and accurately adjust the cut-off of these markers in patients with advanced CKD.

cTnT and cTnI are specific biomarkers of myocardial injury and infarction and correlate with ventricular remodelling after HF and increase as HF progresses and mortality rises. Patients with CKD have elevated cTnT and cTnI levels because of the reduced excretion from the kidney [38], however, in CKD, cardiac troponins can predict cardiovascular and all-cause mortality rates in patients with mild-to-moderate CKD or end-stage renal disease.

CA125, a glycoprotein synthesized by epithelial serosa cells, has gained a lot of attention in recent years. Being widely available for ovarian cancer monitoring, it has emerged as a potential surrogate marker of fluid retention and inflammatory activity in HF. Even more, in Carbohydrate Antigen-125-Guided Therapy in Acute Heart Failure [39] it was successfully used as a diagnostic, prognostic and monitoring biomarker demonstrating a target value of <35 U/mL to reduce adverse events. Interestingly, CA125 appears not significantly influenced by gender, LVEF or renal function, which is particularly appealing in CRS [40].

Inflammatory markers such as C-reactive protein have been shown to predict prognosis both in cardiac and renal dysfunction independently from traditional cardiovascular risk factors [41, 42], and although it is linked to prognosis in HFpEF, this association might be comorbidity driven [43]. The endothelium also plays an important role in CRS, and given the close link between microvascular disease in HF and CKD, endothelial peptides as a vascular endothelial growth factor, platelet-derived growth factor and soluble vascular endothelial growth factor receptors-1 are elevated in HF and CKD [44].

Soluble suppression of tumorigenicity 2 (sST2) is the circulating form of the cellular receptor ST2L, expressed by cardiomyocytes and vascular endothelial cells together with its ligand interleukin-33 (IL-33) after cardiovascular injury. It is a biomarker of cardiomyocyte stress and fibrosis, which provides incremental value to NP for risk stratification of patients across a wide spectrum of cardiovascular diseases [45, 46]. Apparently, sST2 is able to predict cardiovascular events being less affected by GFR and age and with a good performance in HFpEF, correlating with a cardiac remodelling in CKD patients. Added to NP, it has been shown to better stratify the risk of CV events and/or death in CKD patients. The good performance of sST2 independently of GFR seems an attractive feature [47].

Galectin-3 is a protein that in humans is encoded by the *LGALS3* gene that has been intensively studied in HF, as might reflect inflammation and cardiac fibrosis, but seems to be too influenced by renal dysfunction, which reduces its performance in CRS [48, 49].

Focussing on kidneys, as GFR estimating equations based on creatinine have various limitations, with a nonlinear correlation, difficulties in detecting slight alterations in GFR and influence of several confounding non-renal factors (age, race, sex, muscle mass and medication), new biomarkers are needed for the early detection of worsening renal function.

Cystatin C, an inhibitor of cysteine protease produced by nuclear cells, is completely filtered and reabsorbed by the kidneys. It is not affected by age, sex, race or muscle volume, and can serve as a better biomarker of AKI than creatinine [50]. Also, Cystatin C is related to not only HF progression but also cardiovascular events and deaths, independently of renal function, in patients with HF [51, 52, 53]. The combination of Cystatin with NP seems promising as a better stratification and prognosis tool [54, 55].

Neutrophil gelatinase-associated lipocalin (NGAL), kidney injury molecule-1 (KIM-1), N-acetyl-beta-d-glucosaminidase (NAG) and IL-18, identify with high specificity ischaemic and nephrotoxic AKI and not prerenal azotaemia [1]. Nevertheless, the heterogeneous findings and their insufficient performance in isolation suggest that these last biomarkers do not represent a class of interchangeable tubular injury markers [56] and only one biomarker might just not be enough.

As every biomarker has its strengths and weakness, it seems that a combined approach might be reasonable, although the optimal combination needs still to be defined. In the future, it is crucial that a good biomarker or a combination of them would be capable of offering information in terms of the diagnosis, therapy and prognosis of CRS. Table 1 provides a summary of the principal mentioned biomarkers.

**Table 1:** . Summary of the principal biomarkers’ pros and cons in heart failure with preserved ejection fraction and chronic kidney disease.

Biomarker	Advantages	Disadvantages	Evidence
BNP and NTproBNP	• Stabilized diagnostic and prognostic value• Might aid guiding therapy• Widely available• Large evidence from clinical trials as a selection criteria for HF therapy	• Might be elevated in CKD regardless of HF status• Less sensible in HFpEF	+++
CA125	• Diagnostic and prognostic value• Might aid guiding therapy• Not influenced by GFR or LVEF• Widely available	• Cut-off not clearly stablished• HF studies generally based in NP	++/+++
Troponin I and T	• Predictive of cardiovascular events	• Might be elevated in CKD regardless of HF status• Not recommended for HF management	+
sST2	• Incremental value to NP for risk stratification• Less affected from GFR and age than NP	• Not available in many centres• Remains in investigational field	+
Creatinine	• Reflects eGFR• Strong evidence for CKD progression• Prognostic value in HF• Widely available	• Not sensible for slight alterations in GFR• Influenced by several non-renal factors	+++
Cystatin C	• Better biomarker of AKI than creatinine• Predictive of CV events and HF regardless of renal function	• Not available in many centres	++

Evidence scale reflects a personal opinion from authors.

### Pulmonary ultrasound

The use of pulmonary ultrasound to assess the presence of congestion in HF based on line B artefacts has expanded notoriously, given its fast learning curve, the possibility to perform it with any echo device and transducer, and its high sensitivity and specificity [57]. The number of B-lines has been found to be a good indicator of the presence of extravascular lung water, allows identification of HF patients with a worse prognosis and reflects dynamic changes in pulmonary water content after diuretic administration. It has been tested as a guidance tool in randomized trials demonstrating a reduction in the number of HF decompensations without triggering more worsening renal function events [58, 59], and CKD [60].

### Inferior vena cava echo assessment

The diameter of the inferior vena cava (IVC) and its variation with respiration reflects the elasticity of this capacitance vessel. Intrathoracic pressure decreases during inspiration, thereby increasing venous return and causing the collapse of the IVC [61, 62]. In acutely decompensated HF, volume overload dilates the IVC and respirations produce only minimal changes in IVC diameter. An IVC >21 mm is a predictor of adverse outcomes and worsening renal function and is a valuable tool to assess rapidly and noninvasively the volaemia [63, 64].

### Pulmonary artery pressure sensors and other device-driven analysis

Increased filling pressures gradually increase before overt clinical decompensation. In the CardioMEMS Heart Sensor Allows Monitoring of Pressure to Improve Outcomes in NYHA Class III Heart Failure Patients trial [65], HF treatment guided by pulmonary artery pressured by CardioMEMS sensor was associated with a reduction in HF hospitalization compared with clinical management. A target diastolic blood pressure of 20 mmHg helped in the dosing of diuretic and vasodilators, avoiding both congestion and hypovolaemia leading to worsening renal function. Although it is not widely available, this could be a valuable tool to assess volume status in patients with HFpEF and frequent decompensations.

Implantable electronic devices are frequently used in HF. Some of them now include algorithms to estimate thoracic fluid content, which can help to predict HF decompensation early [66], but still remain in investigation. There is also a general agreement suggesting that bioimpedance vector analysis and bioreactance measures may contribute to a better definition of the patient's hydration status [1, 67] and can predict mortality in CKD [68], however its introduction in daily clinical practice is scarce and still remains in investigational fields.

### Intrarenal haemodynamic evaluation by Doppler ultrasonography

Renal congestion constitutes an important mechanism of worsening renal function in patients with CRS. Intrarenal resistive index (RI) and intrarenal venous flow (IRVF) are parameters widely used for assessing renal function in different renal diseases [69]. Doppler ultrasonography as a non‐invasive method to evaluate IRVF in acute HF is a useful tool to evaluate renal congestion. Three patterns of IRVF have been described in the Doppler of interlobar veins: continuous, biphasic and discontinuous. Discontinuous IRVF patterns but no RI are associated with an increase in right atrium pressure and adverse clinical outcome defined as death from cardiovascular disease or unplanned hospitalization for HF [70]. In a study that evaluated the variations in IRVF signals after volume loading and after diuretics treatment in stable HF patients and control subjects, the continuous IRVF pattern was the most common pattern found in both groups. However, after fluid administration, most HF patients developed a biphasic pattern in renal Doppler ultrasound. After diuretic treatment, 70% of HF patients returned to the baseline situation (continuous flow pattern), indicating the recovery of the renal congestion [71].

Taken together, IRVF patterns assessed by renal Doppler ultrasound in intrarenal veins may have a role in identifying the renal haemodynamic disturbances in patients with HF. Biphasic and monophasic discontinuous IRVF may be associated with a phenotype of renal congestion in which an aggressive diuretic treatment would be useful to improve renal function. On the contrary, the presence of a continuous venous flow pattern indicates a normal renal venous pressure, indicating that diuretic needs are lower in these patients. Renal Doppler ultrasound may constitute a tool for monitoring the intrarenal haemodynamic changes in response to treatment [72].

## MANAGEMENT OF CRS

**FIGURE 3:**
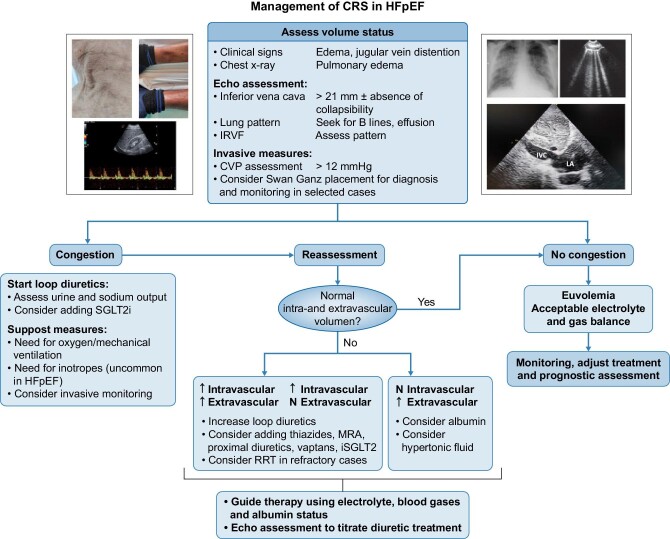
Suggested algorithm for CRS treatment. HFpEF, heart failure with preserved ejection fraction; CRS, cardiorenal syndrome; IRVF, intrarenal venous flow; CVP, central venous pressure; SGLTi, sodium-glucose co-transporter 2 inhibitors; MRA, mineralocorticoid receptor antagonists; RRT, renal replacement therapy; IVC, inferior vena cava; LA, left atria.

Several randomized clinical trials have been centred on the management of HF and CKD, however there is a gap in the knowledge in the management of the CRS. Two main strategies have been developed for its treatment (mainly focussed on CRS type 1): (i) strategies to relieve congestion: mainly based on diuretics and ultrafiltration; and (ii) strategies to improve cardiac output: neurohormonal modulation, vasodilator, inotropic therapy, RAAS inhibition in chronic CRS (angiotensin-converting inhibitors, angiotensin receptor blockers, mineralocorticoids antagonists, beta-blockers and the new antidiabetic drugs). A therapeutic algorithm for CRS is offered in Fig. 3.

Currently, diuretics continue to be the drug of choice for the initial treatment of patients with type 1 CRS. However, they have not been demonstrated to improve hard cardiac endpoints in monotherapy. They are useful for decreasing fluid retention and peripheral oedema as a clinical sign of congestion. Aggressive treatment with diuretics in patients with acute decompensated HF has been demonstrated to improve renal function in type 1 and type II CRS. Patients with HF often develop diuretic resistance during the evolution of their disease, making congestion notoriously challenging to manage and giving them a worse prognosis in terms of survival. In patients with obvious criteria for resistance to diuretics, the only alternative to improve water and salt retention is ultrafiltration. Ultrafiltration can be achieved through two methods, using extracorporeal circulation during haemodialysis (HD), or peritoneal dialysis (PD). Extracorporeal ultrafiltration has been tested in several studies for years, however, it has not demonstrated to increase the survival in CRS patients. In early 2012, Bart *et al.* [73] demonstrated in a randomized clinical trial in hospitalized patients for acute decompensated HF, worsened renal function and persistent congestion, that the use of a stepped pharmacologic-therapy algorithm was superior to a strategy of ultrafiltration in terms of the preservation of renal function at 96 h, with a similar amount of weight loss with the two approaches. Surprisingly, ultrafiltration was associated with a higher rate of adverse events. In concordance with this study, a substudy of the CARRESS-HF (Cardiorenal Rescue Study in Acute Decompensated Heart Failure) that examined differential outcomes of high versus low fluid removal and preserved versus reduced ejection, demonstrated that in patients with HFpEF, ultrafiltration was associated with worsening renal function irrespective of fluid removal rate and higher initial fluid removal was associated with higher rates of adverse clinical outcomes, highlighting variable responses to decongestive therapy. For that reason, patients with HFpEF and resistance to diuretics and other pharmacological drugs have clinical benefits in terms of decreasing hospitalization rates with PD treatment [74]. PD is a home-based treatment that allows a gentle removal of the excess water and sodium with minimal haemodynamic changes, with a great capacity to be able to adapt the requirements according to the clinical situation and the lifestyle of the patients. Icodextrin is a glucose polymer with minimal peritoneal absorption that produces an osmotic gradient that allows significant sodium elimination during PD exchange.

The beneficial effects of the use of PD in patients with HF are related to adequate decongestion, which improves functional class, the rate of hospital admissions and a reduction in hospital days, which has a notable impact on a better quality of life [75]. Despite all these beneficial effects of PD, there are clinical contraindications such as inflammatory bowel disease, ostomy patients and large abdominal surgeries. In addition, there are sociocultural contraindications such as patients with housing problems or patients without family or social support.

To our knowledge, there has been little evidence for effective therapy in HFpEF that improves cardiorenal outcomes such as cardiovascular mortality or HF hospitalizations. In the Prospective Comparison of ARNI with ARB Global Outcomes in HF with Preserved Ejection Fraction randomized clinical trial (efficacy and safety of LCZ696 compared with valsartan, on morbidity and mortality in HFpEF patients), sacubitril-valsartan failed to reduce the rate of total hospitalizations for HF and death from cardiovascular causes among patients of HFpEF [76]. Last year, the Empagliflozin Outcome Trial in Patients with Chronic Heart Failure with Preserved Ejection Fraction (EMPEROR-Preserved) demonstrated the beneficial effect of empagliflozin in terms of reduction of the combined risk of cardiovascular death or hospitalization for HF in patients with HFpEF. The beneficial effects of empagliflozin appeared consistent in patients with or without diabetes. The total number of hospitalizations for HF was lower in the empagliflozin group than in the placebo group [407 with empagliflozin and 541 with placebo; hazard ratio (HR), 0.73; 95% confidence interval (CI), 0.61–0.88; *P* < .001] [77]. Taking these results together with the positive effects demonstrated by the sodium-glucose cotransporter 2 (SGLT2) inhibitors at the renal level in terms of decreasing the combined renal outcome, these drugs seem to be promising for treating patients with CRS [78]. Other large trials focussed on patients with HFpEF such as the Study to Evaluate the Efficacy and Safety of Finerenone on Morbidity & Mortality in Participants With Heart Failure and Left Ventricular Ejection Fraction (FINEARTS-HF, NCT04435626) and a study with Tirzepatide (LY3298176) in Participants With Heart Failure With Preserved Ejection Fraction and Obesity (SUMMIT, NCT 04847557) are currently underway. Hence this point will be extensively discussed in the next section, as patients with CRS need multidisciplinary care to minimize the number of health care visits and their management [79].

## RATIONALE FOR A CARDIORENAL UNIT

Cardiovascular disease, especially HF, is a common thread between nephrologists and cardiologists. This relationship was for the first time described by Robert Bright in 1836 when he observed cardiac structural changes in patients with advanced kidney disease [29]. Since then, numerous advances have been made in summarizing the cardiorenal link in terms of haemodynamic phenotypes, pathophysiology, therapeutic options and clinical outcomes.

Mechanisms involved in the kidney–heart cross-talk are complex, involving different haemodynamic and non-haemodynamic pathways, which allows targeting different therapeutic strategies. In this regard, a multidisciplinary approach is fundamental. A strong ‘marriage’ of cardiologists and nephrologists enhances patient-tailored decisions with a wide vision based on the characteristics of each case. A close working relationship between nephrologists and cardiologists is the key to successfully managing symptoms and prolonging life where possible; treating with diuretics, angiotensin-converting enzyme inhibitors and mineralocorticoid receptor antagonists, while avoiding electrolyte abnormalities and AKI; and multidisciplinary team decisions for dialysis and device therapy, altogether, maybe the key for the CRS treatment [80]. It has been demonstrated that to achieve appropriate therapy and the best possible outcome, combined cardiology–nephrology clinics are needed [80]. In addition, these new multidisciplinary strategies are welcomed by patients, given that with a single visit they receive excellent, complete and coherent feedback regarding pharmacologic therapy, intravenous iron administration, congestion management, education and device therapies. The main problem is that it requires proper resources, including the presence of a nephrologist, cardiologist, nurse and an appropriate shared location [79]. However, as the multidisciplinary strategy might lead to a decreased number of visits and hospitalization rate, a huge economic benefit for the health system should also be expected. More studies promoted by cardiologist and nephrologist societies are needed to ascertain the evidence of cardiorenal unit benefits in this group of patients with CRS.

## CONCLUSIONS

HF is a major clinical and public health problem and HFpEF is the most common form of HF. CKD has also a high global prevalence, and renal dysfunction is one of the most important risk factors predicting mortality and poor outcomes in HF patients. Therefore, understanding the interrelation between the heart and kidneys in the clinical scenario of CRS is essential. We currently have new tools that help us in the diagnosis, such as biomarkers and imaging techniques. Altogether this allows the early identification of these patients, with the aim of offering them the best treatment. The appearance of new drugs, such as SGLT2 inhibitors, represents a start for the prognostic improvement of CRS. The complexity of these patients requires multidisciplinary management and the implementation of cardiorenal units, which are expected to reduce the number of visits as well as the hospitalization rate, with a positive impact not only on the patient but also on the health system.

## References

[1] Ronco C , McCullough P, Anker SD et al. Cardio-renal syndromes: report from the consensus conference of the acute dialysis quality initiative. Eur Heart J 2010; 31: 703–711 20037146 10.1093/eurheartj/ehp507 PMC2838681

[2] Afsar B , Ortiz A, Covic A et al. Focus on renal congestion in heart failure. Clin Kidney J 2016; 9: 39–47 26798459 10.1093/ckj/sfv124 PMC4720202

[3] González AO , de Francisco A, Gayoso P et al. Prevalence of chronic renal disease in Spain: Results of the EPIRCE study. Nefrologia 2010; 30: 78–86 20038967 10.3265/Nefrologia.pre2009.Dic.5732

[4] Gorostidi M , Sánchez-Martínez M, Ruilope LM et al. Prevalencia de enfermedad renal crónica en España: impacto de la acumulación de factores de riesgo cardiovascular. Nefrología 2018; 38: 606–615 29914761 10.1016/j.nefro.2018.04.004

[5] Hill NR , Fatoba ST, Oke JL et al. Global prevalence of chronic kidney disease—a systematic review and meta-analysis. PLoS One 2016; 11: e0158765 27383068 10.1371/journal.pone.0158765 PMC4934905

[6] Jager KJ , Kovesdy C, Langham R et al. A single number for advocacy and communication—Worldwide more than 850 million individuals have kidney diseases. Kidney Int, 2019; 96: 1048–1050 31582227 10.1016/j.kint.2019.07.012

[7] Thompson S , James M, Wiebe N et al. Cause of death in patients with reduced kidney function. J Am Soc Nephrol 2015; 26: 2504–2511 25733525 10.1681/ASN.2014070714 PMC4587695

[8] Bock JS , Gottlieb SS. Cardiorenal syndrome: new perspectives. Circulation 2010; 121: 2592–2600 20547939 10.1161/CIRCULATIONAHA.109.886473

[9] Damman K , Valente MAE, Voors AA et al. Renal impairment, worsening renal function, and outcome in patients with heart failure: an updated meta-analysis. Eur Heart J 2014; 35: 455–469 24164864 10.1093/eurheartj/eht386

[10] Lippi G , Sanchis-Gomar F. Global epidemiology and future trends of heart failure. AME Med J 2020; 5: 1–6

[11] Park M , Hsu CY, Li Y et al. Associations between kidney function and subclinical cardiac abnormalities in CKD. J Am Soc Nephrol 2012; 23: 1725–1734 22935481 10.1681/ASN.2012020145 PMC3458463

[12] Tuegel C , Bansal N. Heart failure in patients with kidney disease. Heart 2017; 103: 1848–1853 28716974 10.1136/heartjnl-2016-310794

[13] Saran R , Robinson B, Abbott KC et al. US Renal Data System 2016 Annual Data Report: Epidemiology of Kidney Disease in the United States. Am J Kidney Dis 2017; 69: A7–A8 28236831 10.1053/j.ajkd.2016.12.004 PMC6605045

[14] Foley RN . Clinical epidemiology of cardiac disease in dialysis patients: left ventricular hypertrophy, ischemic heart disease, and cardiac failure. Semin Dial 2003; 16: 111–117 12641874 10.1046/j.1525-139x.2003.160271.x

[15] Hillege HL , Nitsch D, Pfeffer MA et al. Renal function as a predictor of outcome in a broad spectrum of patients with heart failure. Circulation 2006; 113: 671–678 16461840 10.1161/CIRCULATIONAHA.105.580506

[16] Lazzeri C , Valente S, Tarquini R et al. Cardiorenal syndrome caused by heart failure with preserved ejection fraction. Intern J Nephrol 2011; 24: 421–437 10.4061/2011/634903 PMC3038429 21331316

[17] McDonagh TA , Metra M, Adamo M et al. 2021 ESC Guidelines for the diagnosis and treatment of acute and chronic heart failure. Eur Heart J 2021; 42: 3599–3726 34447992 10.1093/eurheartj/ehab368

[18] Reddy YNV , Carter RE, Obokata M et al. A simple, evidence-based approach to help guide diagnosis of heart failure with preserved ejection fraction. Circulation 2018; 138: 861–870 29792299 10.1161/CIRCULATIONAHA.118.034646 PMC6202181

[19] Pieske B , Tschöpe C, De Boer RA et al. How to diagnose heart failure with preserved ejection fraction: the HFA-PEFF diagnostic algorithm: a consensus recommendation from the Heart Failure Association (HFA) of the European Society of Cardiology (ESC). Eur Heart J 2019; 40: 3297–3317 31504452 10.1093/eurheartj/ehz641

[20] Task A , Members F, Mcdonagh TA et al. 2021 ESC Guidelines for the diagnosis and treatment of acute and chronic heart failure. Eur Heart J 2021; 36: 1–128 10.1093/eurheartj/ehab368 34447992

[21] Kumar U , Wettersten N, Garimella PS. Cardiorenal syndrome: pathophysiology. Cardiol Clin 2019; 37: 251–265 31279419 10.1016/j.ccl.2019.04.001 PMC6658134

[22] McDonald K . Diastolic heart failure in the elderly: underlying mechanisms and clinical relevance. Int J Cardiol 2008; 125: 197–202 18160154 10.1016/j.ijcard.2007.10.002

[23] Borlaug BA . The pathophysiology of heart failure with preserved ejection fraction. Nat Rev Cardiol 2014; 11: 507–515 24958077 10.1038/nrcardio.2014.83

[24] Zile MR , Baicu CF, Gaasch WH. Diastolic heart failure—abnormalities in active relaxation and passive stiffness of the left ventricle. N Engl J Med 2004; 350: 1953–1959 15128895 10.1056/NEJMoa032566

[25] Van Aelst LNL , Arrigo M, Placido R et al. Acutely decompensated heart failure with preserved and reduced ejection fraction present with comparable haemodynamic congestion. Eur J Heart Fail 2018; 20: 738–747 29251818 10.1002/ejhf.1050

[26] Nohria A , Hasselblad V, Stebbins A et al. Cardiorenal Interactions. Insights from the ESCAPE trial. J Am Coll Cardiol 2008; 51: 1268–1274 18371557 10.1016/j.jacc.2007.08.072

[27] Mullens W , Abrahams Z, Francis G et al. Importance of venous congestion for worsening of renal function in advanced decompensated heart failure. Eur J Heart Fail Suppl 2008; 53: 589–596 10.1016/j.jacc.2008.05.068 PMC2856960 19215833

[28] Merrill AJ . Edema and decreased renal blood flow in patients with chronic congestive heart failure; evidence of forward failure as the primary cause of edema. J Clin Invest 1946; 25: 389–400 20986045

[29] Rangaswami J , Bhalla V, Blair JEA et al. Cardiorenal syndrome: classification, pathophysiology, diagnosis, and treatment strategies: a scientific statement from the American Heart Association. Circulation 2019; 139: e840–e878 30852913 10.1161/CIR.0000000000000664

[30] Giam B , Kaye DM, Rajapakse NW. Role of renal oxidative stress in the pathogenesis of the cardiorenal syndrome. Heart Lung Circ 2016; 25: 874–880 27132623 10.1016/j.hlc.2016.02.022

[31] Shah SJ , Lam CSP, Svedlund S et al. Prevalence and correlates of coronary microvascular dysfunction in heart failure with preserved ejection fraction: pROMIS-HFpEF. Eur Heart J 2018; 39: 3439–3450 30165580 10.1093/eurheartj/ehy531 PMC6927847

[32] Agrawal A , Naranjo M, Kanjanahattakij N et al. Cardiorenal syndrome in heart failure with preserved ejection fraction—an under-recognized clinical entity. Heart Fail Rev 2019; 24: 421–437 31127482 10.1007/s10741-018-09768-9

[33] Nickel NP , O'Leary JM, Brittain EL et al. Kidney dysfunction in patients with pulmonary arterial hypertension. Pulmonary Circulation 2017; 7: 38–54 28680564 10.1086/690018 PMC5448543

[34] Borlaug BA , Melenovsky V, Russell SD et al. Impaired chronotropic and vasodilator reserves limit exercise capacity in patients with heart failure and a preserved ejection fraction. Circulation 2006; 114: 2138–2147 17088459 10.1161/CIRCULATIONAHA.106.632745

[35] Palau P , Seller J, Domínguez E et al. Beta-blockers withdrawal in patients with heart failure with preserved ejection fraction and chronotropic incompetence: effect on functional capacity rationale and study design of a prospective, randomized, controlled trial (The Preserve-HR trial). Clin Cardiol 2020; 43: 423–429 32073676 10.1002/clc.23345 PMC7244302

[36] Fu S , Zhao S, Ye P, Luo L. Biomarkers in cardiorenal syndromes. Biomed Res Int 2018; 2018: 1–8 10.1155/2018/9617363 PMC5859843 29693019

[37] Maisel A , Mueller C, Adams K et al. State of the art: using natriuretic peptide levels in clinical practice. Eur J Heart Fail 2008; 10: 824–839 18760965 10.1016/j.ejheart.2008.07.014

[38] Yang H , Liu J, Luo H et al. Improving the diagnostic accuracy of acute myocardial infarction with the use of high-sensitive cardiac troponin T in different chronic kidney disease stages. Sci Rep 2017; 7: 1–7 28145489 10.1038/srep41350 PMC5286511

[39] Núñez J , Llàcer P, Bertomeu-González V et al. Carbohydrate Antigen-125–Guided Therapy in Acute Heart Failure: CHANCE-HF: A Randomized Study. JACC Heart Fail 2016; 4: 833–843 27522630 10.1016/j.jchf.2016.06.007

[40] Llàcer P , Bayés-Genís A, Núñez J. Carbohydrate antigen 125 in heart failure. New era in the monitoring and control of treatment. Medicina Clínica 2019; 152: 266–273 30442374 10.1016/j.medcli.2018.08.020

[41] Kang S , Fan L-Y, Chen M et al. Relationship of high-sensitivity C-Reactive protein concentrations and systolic heart failure. Curr Vasc Pharmacol 2017; 15: 390–396 28393707 10.2174/1570161115666170404121619

[42] Li W , Xiong L, Fan L et al. Association of baseline, longitudinal serum high-sensitive C-reactive protein and its change with mortality in peritoneal dialysis patients. BMC Nephrol 2017; 18: 1–10 28676043 10.1186/s12882-017-0624-4 PMC5496342

[43] DuBrock HM , AbouEzzeddine OF, Redfield MM. High-sensitivity C-reactive protein in heart failure with preserved ejection fraction. PLoS One 2018; 13: 1–16 10.1371/journal.pone.0201836 PMC6095520 30114262

[44] Vorovich E , French B, Ky B et al. Biomarker predictors of cardiac hospitalization in chronic heart failure: a recurrent event analysis. J Card Fail 2014; 20: 569–576 24929121 10.1016/j.cardfail.2014.05.013 PMC4142349

[45] Manzano-Fernndez S , Mueller T, Pascual-Figal D et al. Usefulness of soluble concentrations of interleukin family member ST2 as predictor of mortality in patients with acutely decompensated heart failure relative to left ventricular ejection fraction. Am J Cardiol 2011; 107: 259–267 21211603 10.1016/j.amjcard.2010.09.011 PMC3218083

[46] Ky B , French B, McCloskey K et al. High-sensitivity ST2 for prediction of adverse outcomes in chronic heart failure. Circ Heart Fail 2011; 4: 180–187 21178018 10.1161/CIRCHEARTFAILURE.110.958223 PMC3163169

[47] Plawecki M , Morena M, Kuster N et al. sST2 as a new biomarker of chronic kidney disease-induced cardiac remodeling: impact on risk prediction. Mediators Inflamm 2018; 2018. 10.1155/2018/3952526 PMC6196921 30402040

[48] Zamora E , Lupón J, De Antonio M et al. Renal function largely influences Galectin-3 prognostic value in heart failure. Int J Cardiol 2014; 177: 171–177 25499371 10.1016/j.ijcard.2014.09.011

[49] Besler C , Lang D, Urban D et al. Plasma and cardiac galectin-3 in patients with heart failure reflects both inflammation and fibrosis: implications for its use as a biomarker. Circ Heart Fail 2017; 10: 1–9 10.1161/CIRCHEARTFAILURE.116.003804 28288987

[50] Roos JF , Doust J, Tett SE et al. Diagnostic accuracy of cystatin C compared to serum creatinine for the estimation of renal dysfunction in adults and children—A meta-analysis. Clin Biochem 2007; 40: 383–391 17316593 10.1016/j.clinbiochem.2006.10.026

[51] Lassus JPE , Nieminen MS, Peuhkurinen K et al. Markers of renal function and acute kidney injury in acute heart failure: definitions and impact on outcomes of the cardiorenal syndrome. Eur Heart J 2010; 31: 2791–2798 20801926 10.1093/eurheartj/ehq293

[52] Rafouli-Stergiou P , Parissis J, Farmakis D et al. Prognostic value of in-hospital change in cystatin C in patients with acutely decompensated heart failure and renal dysfunction. Int J Cardiol 2015; 182: 74–76 25576725 10.1016/j.ijcard.2014.12.135

[53] Jang SY , Yang DH, Kim HJ et al. Prognostic value of cystatin C-derived estimated glomerular filtration rate in patients with acute heart failure. Cardiorenal Med 2020; 10: 232–242 32316021 10.1159/000504084

[54] Alehagen U , Dahlström U, Lindahl TL. Cystatin C and NT-proBNP, a powerful combination of biomarkers for predicting cardiovascular mortality in elderly patients with heart failure: results from a 10-year study in primary care. Eur J Heart Fail 2009; 11: 354–360 19228797 10.1093/eurjhf/hfp024

[55] Truong QA , Szymonifka J, Januzzi JL et al. Cardiorenal status using amino-terminal pro–brain natriuretic peptide and cystatin C on cardiac resynchronization therapy outcomes: from the BIOCRT Study. Heart Rhythm 2019; 16: 928–935 30590191 10.1016/j.hrthm.2018.12.023 PMC6545247

[56] Testani JM , Tang WHW. Biomarkers of acute kidney injury in chronic heart failure: what do the signals mean? JACC Heart Fail 2013; 1: 425–426 24621974 10.1016/j.jchf.2013.08.001 PMC12101720

[57] Price S , Platz E, Cullen L et al. Expert consensus document: echocardiography and lung ultrasonography for the assessment and management of acute heart failure. Nat Rev Cardiol 2017; 14: 427–440 28447662 10.1038/nrcardio.2017.56 PMC5767080

[58] Araiza-Garaygordobil D , Gopar-Nieto R, Martinez-Amezcua P et al. A randomized controlled trial of lung ultrasound-guided therapy in heart failure (CLUSTER-HF study). Am Heart J 2020; 227: 31–39 32668323 10.1016/j.ahj.2020.06.003

[59] Rivas-Lasarte M , Álvarez-García J, Fernández-Martínez J et al. Lung ultrasound-guided treatment in ambulatory patients with heart failure: a randomized controlled clinical trial (LUS-HF study). Eur J Heart Fail 2019; 21: 1605–1613 31667987 10.1002/ejhf.1604

[60] Covic A , Siriopol D, Voroneanu L. Use of lung ultrasound for the assessment of volume status in CKD. Am J Kidney Dis 2018; 71: 412–422 29274919 10.1053/j.ajkd.2017.10.009

[61] Guiotto G , Masarone M, Paladino F et al. Inferior vena cava collapsibility to guide fluid removal in slow continuous ultrafiltration: a pilot study. Intensive Care Med 2010; 36: 692–696 20094880 10.1007/s00134-009-1745-4

[62] Costanzo MR . The cardiorenal syndrome in heart failure. Heart Fail Clin 2020; 16: 81–97 31735318 10.1016/j.hfc.2019.08.010

[63] Lee HF , Hsu LA, Chang CJ et al. Prognostic significance of dilated inferior vena cava in advanced decompensated heart failure. Int J Cardiovasc Imaging 2014; 30: 1289–1295 24939288 10.1007/s10554-014-0468-y

[64] Blehar DJ , Dickman E, Gaspari R. Identification of congestive heart failure via respiratory variation of inferior vena cava diameter. Am J Emerg Med 2009; 27: 71–75 19041537 10.1016/j.ajem.2008.01.002

[65] Abraham WT , Adamson PB, Bourge RC et al. Wireless pulmonary artery haemodynamic monitoring in chronic heart failure: a randomised controlled trial. Lancet North Am Ed 2011; 377: 658–666 10.1016/S0140-6736(11)60101-3 21315441

[66] Boehmer JP , Hariharan R, Devecchi FG et al. A multisensor algorithm predicts heart failure events in patients with implanted devices: results from the MultiSENSE Study. JACC Heart Fail 2017; 5: 216–225 28254128 10.1016/j.jchf.2016.12.011

[67] Hankinson SJ , Williams CH, Ton VK et al. Should we overcome the resistance to bioelectrical impedance in heart failure? Expert Rev Med Devices 2020; 17: 785–794 32658589 10.1080/17434440.2020.1791701 PMC8356137

[68] Wizemann V , Wabel P, Chamney P et al. The mortality risk of overhydration in haemodialysis patients. Nephrol Dial Transplant 2009; 24: 1574–1579 19131355 10.1093/ndt/gfn707 PMC2668965

[69] Di Nicolò P , Granata A. Renal intraparenchymal resistive index: the ultrasonographic answer to many clinical questions. J Nephrol 2019; 32: 527–538 30539416 10.1007/s40620-018-00567-x

[70] Iida N , Seo Y, Sai S et al. Clinical implications of intrarenal hemodynamic evaluation by doppler ultrasonography in heart failure. JACC Heart Fail 2016; 4: 674–682 27179835 10.1016/j.jchf.2016.03.016

[71] Nijst P , Martens P, Dupont M et al. Intrarenal flow alterations during transition from euvolemia to intravascular volume expansion in heart failure patients. JACC Heart Fail 2017; 5: 672–681 28711449 10.1016/j.jchf.2017.05.006

[72] de la Espriella-Juan R , Núñez E, Miñana G et al. Intrarenal venous flow in cardiorenal syndrome: a shining light into the darkness. ESC Heart Failure 2018; 5: 1173 10.1002/ehf2.12362 PMC6300820 30295431

[73] Bart BA , Goldsmith SR, Lee KL et al. Ultrafiltration in decompensated heart failure with cardiorenal syndrome. N Engl J Med 2012; 367: 2296–2304 23131078 10.1056/NEJMoa1210357 PMC3690472

[74] Fudim M , Brooksbank J, Giczewska A et al. Ultrafiltration in acute heart failure: implications of ejection fraction and early response to treatment from CARRESS-HF. J Am Heart Assoc 2020; 9: e015752, 33289458 10.1161/JAHA.119.015752 PMC7955382

[75] Chionh CY , Clementi A, Poh CB et al. The use of peritoneal dialysis in heart failure: a systematic review. Perit Dial Int 2020; 40: 527–539 32063182 10.1177/0896860819895198

[76] Solomon SD , McMurray JJV, Anand IS et al. Angiotensin-Neprilysin inhibition in heart failure with preserved ejection fraction. N Engl J Med 2019; 381: 1609–1620 31475794 10.1056/NEJMoa1908655

[77] Anker SD , Butler J, Filippatos G et al. Empagliflozin in heart failure with a preserved ejection fraction. N Engl J Med 2021; 385: 1451–1461 34449189 10.1056/NEJMoa2107038

[78] Fernandez-Fernandez B , Fernandez-Prado R, Górriz JL et al. Canagliflozin and renal events in diabetes with established nephropathy clinical evaluation and study of diabetic nephropathy with atrasentan: what was learned about the treatment of diabetic kidney disease with canagliflozin and atrasentan? Clin Kidney J 2019; 12: 313–321 31198532 10.1093/ckj/sfz070 PMC6543971

[79] de la Espriella R , González M, Górriz JL et al. Setting up a cardiorenal clinic. Consensus document of the Cardiorenal Working Groups of the Spanish Society of Cardiology and the Spanish Society of Nephrology. REC: CardioClinics 2021; 56: 284–295

[80] Banerjee D , Rosano G, Herzog CA. Management of heart failure patient with CKD. Clin J Am Soc Nephrol 2021; 16: 1131–1139 33495289 10.2215/CJN.14180920 PMC8425606

